# Combined enzyme/prodrug treatment by genetically engineered AT-MSC exerts synergy and inhibits growth of MDA-MB-231 induced lung metastases

**DOI:** 10.1186/s13046-015-0149-2

**Published:** 2015-04-09

**Authors:** Miroslava Matuskova, Zuzana Kozovska, Lenka Toro, Erika Durinikova, Silvia Tyciakova, Zuzana Cierna, Roman Bohovic, Lucia Kucerova

**Affiliations:** Laboratory of Molecular Oncology, Cancer Research Institute of Slovak Academy of Sciences, Vlarska 7, Bratislava, 833 91 Slovakia; Institute of Pathological Anatomy, Faculty of Medicine, Comenius University, Sasinkova 4, Bratislava, 813 72 Slovakia

**Keywords:** Mesenchymal stromal cells, Gene therapy, Cytosine deaminase, HSV thymidine kinase, Metastasis, Synergy, Combined treatment

## Abstract

**Background:**

Metastatic spread of tumor cells remains a serious problem in cancer treatment. Gene-directed enzyme/prodrug therapy mediated by tumor-homing genetically engineered mesenchymal stromal cells (MSC) represents a promising therapeutic modality for elimination of disseminated cells. Efficacy of gene-directed enzyme/prodrug therapy can be improved by combination of individual systems. We aimed to define the combination effect of two systems of gene therapy mediated by MSC, and evaluate the ability of systemically administered genetically engineered mesenchymal stromal cells to inhibit the growth of experimental metastases derived from human breast adenocarcinoma cells MDA-MB-231/EGFP.

**Methods:**

Human adipose tissue-derived mesenchymal stromal cells (AT-MSC) were retrovirally transduced with fusion yeast *cytosine deaminase::uracil phosphoribosyltransferase* (CD::UPRT) or with *Herpes simplex virus* thymidine kinase (HSVtk). Engineered MSC were cocultured with tumor cells in the presence of prodrugs 5-fluorocytosin (5-FC) and ganciclovir (GCV). Combination effect of these enzyme/prodrug approaches was calculated. SCID/bg mice bearing experimental lung metastases were treated with CD::UPRT-MSC, HSVtk-MSC or both in combination in the presence of respective prodrug(s). Treatment efficiency was evaluated by EGFP-positive cell detection by flow cytometry combined with real-time PCR quantification of human cells in mouse organs. Results were confirmed by histological and immunohistochemical examination.

**Results:**

We demonstrated various extent of synergy depending on tested cell line and experimental setup. The strongest synergism was observed on breast cancer-derived cell line MDA-MB-231/EGFP. Systemic administration of CD::UPRT-MSC and HSVtk-MSC in combination with 5-FC and GCV inhibited growth of MDA-MB-231 induced lung metastases.

**Conclusions:**

Combined gene-directed enzyme/prodrug therapy mediated by MSC exerted synergic cytotoxic effect and resulted in high therapeutic efficacy *in vivo*.

**Electronic supplementary material:**

The online version of this article (doi:10.1186/s13046-015-0149-2) contains supplementary material, which is available to authorized users.

## Introduction

Mesenchymal stromal cells (MSC) possess the tumor-homing capability which predetermines them as suitable vehicles for delivery of therapeutic molecules into sites of tumor burden [1-3]. Systemic administration of MSC expressing prodrug-converting gene in combination with appropriate prodrug represents one of the promising experimental approaches in cancer treatment. It was demonstrated on glioblastoma, melanoma, prostate, colon and hepatocellular carcinoma model [4-7]. The two most widely used approaches represent *Herpes simplex virus* thymidine kinase (HSVtk) in combination with ganciclovir (GCV) and cytosine deaminase (CD) derived from bacteria or yeast combined with 5-fluorocytosine (5-FC) (alone or fused with uracil phosphoribosyltransferase, UPRT). Similarly to all other treatments, also this therapeutic system is limited. As we demonstrated previously, treatment efficacy can be influenced by expression level of enzymes involved in drug activation/degradation, intercellular communication or by expression of ABC transporters effluxing toxic metabolites out of cells. We have also demonstrated insufficient efficacy of the treatment by adipose tissue-derived MSC (AT-MSC) expressing CD::UPRT combined with 5-FC on adenocarcinoma-derived cell line MDA-MB-231/EGFP *in vitro*. On the other hand, this cell line was sensitive to treatment by HSVtk expressing AT-MSC and GCV [8].

In order to improve the efficiency of the treatment we used the combination of HSVtk/GCV and CD::UPRT/5-FC approaches, however individual groups obtained different to contradictory results after combination of two suicide genes and two prodrugs. Depending on the experimental setup they evaluated the effect as antagonistic, additive or synergic.

Recently, the efficacy of combined enzyme/prodrug treatment mediated by cellular vehicles was reported. Improved efficiency of cell-based CD/HSVtk combined therapy was demonstrated on breast cancer model *in vivo*. Circumtumoral injection of human amniotic fluid-derived stem cells retrovirally transduced with fusion bacterial CD-HSVtk gene resulted in breast tumor suppression maintaining the original breast tissue structures [9]. Intravenously administered neural stem cells (NSC) expressing CD and HSVtk exerted strong bystander effect on brain metastases derived from human lung cancer cells [10]. Potency and safety of double gene therapy mediated by NSC was approved on glioblastoma models [11,12].

In the present study, we demonstrated synergy of the combined MSC-based gene therapy and significant therapeutic effect on experimental lung metastases induced by MDA-MB-231/EGFP cell line.

## Material and methods

### Chemicals

All chemicals were purchased from Sigma (St Louis, MO, USA), if not stated otherwise.

### Cells

Human breast adenocarcinoma cell lines MDA-MB-231 (ATCC® Number HTB-26™), T47D (ATCC Number® HTB-133™) and melanoma cells A375 were cultured in high-glucose (4500 mg/ml) Dulbecco’s modified Eagle medium (DMEM, PAA Laboratories GmbH) supplemented with 7% fetal bovine serum (FBS, Biochrom AG), gentamicine 10 μg/ml, and 2 mM glutamine.

AT-MSC were isolated by plastic adherence technique as described previously [7]. Cells were cultured in low glucose DMEM (1000 mg/ml) (PAA Laboratories GmbH) with GlutamMAX™ (PAA Laboratories GmbH) supplemented with 5% HyClone® AdvanceSTEM™ Mesenchymal Stem Cell Growth Supplement (Thermo Scientific) 5% FCS and gentamicine 10 μg/ml. AT-MSC were characterized by flow cytometry using MSC phenotyping kit (Miltenyi Biotec, Bergisch Gladbach, Germany) and by differentiation as stated elsewhere [7]. Four isolates of AT-MSC were used in this study.

### Evaluation of gap-junctional intercellular communication

GJIC was detected by a fluorescent dye transfer assay evaluated by flow cytometry as described previously [13] with slight modification. Tumor cells were trypsinized, washed twice with PBS and stained with 5 μl of Vybrant DiI (Invitrogen, Carlsbad, CA) in 1 ml PBS per 1×10^6^ cells for 20 min at 37°C. Monolayer of AT-MSC was washed twice with PBS and labelled with 1 μM Calcein AM (Invitrogen Carlsbad, CA) for 20 min at 37°C. Cells were washed three times in PBS, trypsinized and mixed in a ratio 1:1 with T47D, seeded on 24-well plate (100,000 cells/well) for 24 hours coculture.

### Retroviral transduction

Replication-defective retroviral vectors based on Moloney murine leukemia virus (MMLV) were used for gene transfer as described previously [8]. CD::UPRT gene carrying vector pST2 [7] and vector pAPtk coding HSVtk [13] were used for preparation of therapeutic AT-MSC. Construct pAP-EGFP containing gene for enhanced green fluorescent protein (EGFP) [13] was used for transduction of tumor cells.

### Evaluation of the efficacy of combined treatment *in vitro*

The efficacy of the treatment was evaluated by fluorimetric assay as described in [8]. EGFP-expressing tumor cells were cocultured with control AT-MSC (ratio 10:2) or with the mixture of HSVtk-MSC and CD::UPRT-MSC (ratio 10:1:1) in black 96-well plates. The test was performed in high-glucose DMEM supplemented with 7% FCS. After 24 hours the treatment started. At the sequential treatment the 5-FC-containing medium was added. The next day, medium was aspired and replaced by GCV-containing medium. Plates were incubated for next 48 hours. Medium containing both prodrugs was added at the same time at the simultaneous treatment. Cells were cultured for next 72 or 120 hours. Before evaluation, the cells were washed three times with PBS. Measurement was performed in PBS containing 0.2% Nonidet P40. Assay was performed in quadruplicates, results were expressed as % of relative fluorescence (average ± standard deviation). Proliferation of cells in medium without prodrugs was set to 100%. Combination effect of two GDEPT approaches was calculated according to Chou [14].

Briefly, for median-effect plots, log (fa/fu) was plotted against log (D), where fa represents affected fraction (proportion of dead cells), fu unaffected fraction (viable cells), and D represents the concentration of the prodrug(s). Combination index (CI) was computed for every affected fraction (fa): CI < 1 represents synergism, CI = 1 represents additivity, antagonism is defined with CI > 1. Calcusyn software was used for analysis [15].

### Metastases induction and treatment *in vivo*

Six to eight weeks old SCID/bg mice were used in accordance with the institutional guidelines under the approved protocols. Lungs’ xenografts were induced by 1.5×10^6^ MDA-MB-231/EGFP cells resuspended in 100 μl PBS and administered into tail vein. Mice were randomly divided into groups according to the treatment. For evaluation of the ability of AT-MSC to colonize the lung tissue, 1×10^6^ of AT-MSC was fluorescently stained with Vybrant DiI solution (Invitrogen, Carlsbad, CA) in 1 ml of PBS for 20 min at 37°C.

Therapeutic (CD::UPRT-MSC, HSVtk-MSC or mixture of both in ratio 1:1) or control AT-MSC were administered intravenously into tail vein (1×10^6^ cells in 150 μl PBS) 9 days after the injection of the tumor cells. Treated animals received daily dose of 500 mg 5-FC/kg/day i.p (Ancotil, Valeant, Poland) and/or 50 mg/kg/day of GCV (Cymevene, Roche, Montreal, CA) for 14 days. Prodrugs were administered in two injections concomitantly. Mice were sacrificed on day 30–33 after the tumor cells administration, when the control – untreated animals had to be euthanized.

### Detection of tumor cells in lungs by flow cytometry

Lung tissue was homogenized using gentleMACS Dissociator (Miltenyi Biotec) in the presence of 0.05% trypsin-EDTA (Gibco). Suspension was subsequently incubated at 37°C for 20 min and once again homogenized by gentleMACS. The cells were filtered through 30 μm filters, stained with DAPI, or 7AAD and analyzed by BD FACSCantoII™ analyzer equipped with BD FASCDiva™ software. One hundred thousand events were recorded. The FCSExpress software was used for evaluation.

### DNA extraction and qPCR analysis

Genomic DNA was isolated from gentleMACS homogenized lung tissue as stated above. NucleoSpin**®** Tissue isolation kit (Macherey Nagel, Düren, Germany) was used for DNA isolation. We proceeded according to the manufacturer’s instructions. DNA analyses were performed by qPCR in Maxima probe qPCR master mix (Fermentas, Germany) using thermocycler CFX 96 (Bio-Rad, USA). One hundred and fifty ng of purified DNA from tested tissues was amplified using specific primers and probes (Table 1) in duplex quantitative PCR: Pre-treatment 50°C 2 minutes, initial denaturation 95°C 10 minutes followed by 50 cycles at 95°C for 20 seconds, 60°C 1 minute and then the plate was read. Final annealing was at 60°C for 10 minutes followed by cooling step to 7°C.

**Table 1:** **Sequences of primers used in this study**

RAPSYN sense			5′- ACAATGCCATCAACCTTAGC −3′
RAPSYN antisense			5′- GTGAGTGAGGCAGGTTCATT-3′
RAPSYN probe	3′-BHQ-1	5′-JOE	5′- AGAATTATCTGACCCACCCATCCTGC-3′
β –GLOBIN sense			5′- CTAAGCCAGTGCCAGAAGAG-3′
β -GLOBIN antisense			5′- CTCTGCCCTGACTTTTATGC-3′
β GLOBIN probe	3′-BHQ-1	5′-FAM	5′- ACGGCTGTCATCACTTAGACCTCACC-3′

The mouse Rapsyn (receptor-associated protein at the synapse) gene was used as an internal control. The quantity of PCR product was calculated by Ct value. The positive control DNA was isolated from human cells MDA-MB-231/EGFP. DNA isolated from the mouse cell line NIH/3T3 (ATCC® CRL-1658™) was used as a negative control.

Evaluation of human specificity of β-globin amplification was demonstrated using 2-fold dilution for 100 ng to 0 ng human gDNA per assay in PCR grade water and in 0 to 100 ng murine gDNA per assay. The quantity of human gDNA was calculated according to the calibration curve prepared as stated above. The amount of human β-globin was related to 150 ng of total DNA.

### Histology and immunohistochemistry

Formalin fixed, paraffin embedded lung tissues were cut into 5 μm thick sections, stained with hematoxylin/eosin and evaluated by light microscopy. Immunohistochemical staining was performed to detect EGFP expressed in tumor cells. Slides were deparaffinized and rehydrated in phosphate buffered saline solution (10 mM, pH 7.2). The tissue epitopes were demasked using the automated water bath heating process in Dako PT Link (Dako, Glostrup, Denmark); the slides were incubated in pH 6.0 citrate retrieval buffer at 98°C for 20 minutes. The slides were subsequently incubated for 60 minutes at room temperature with the primary rabbit polyclonal antibody against GFP (Abcam, anti-GFP, ab290) diluted 1:500 in Dako REAL antibody diluent (Dako, Glostrup, Denmark) and immunostained using anti-mouse/anti-rabbit immuno-peroxidase polymer (EnVision FLEX/HRP, Dako, Glostrup, Denmark) for 30 minutes at room temperature, according to the manufacturer’s instructions. For visualization, the slides were reacted with diaminobenzidine substrate-chromogen solution (DAB, Dako, Glostrup, Denmark) for 5 minutes. Finally, the slides were counterstained with hematoxylin.

### Statistical analysis

The Mann Whitney test (comparison of two groups) and Kruskal-Wallis test (comparison of three groups) were used for statistical analysis. The data were analyzed by GraphPad Prism® software (LA Jolla, CA). The value of p < 0.05 was considered statistically significant.

## Results

### Sequential treatment by combined cell-based GDEPT exerts synergic effect on tumor cells *in vitro*

**Figure 1:**
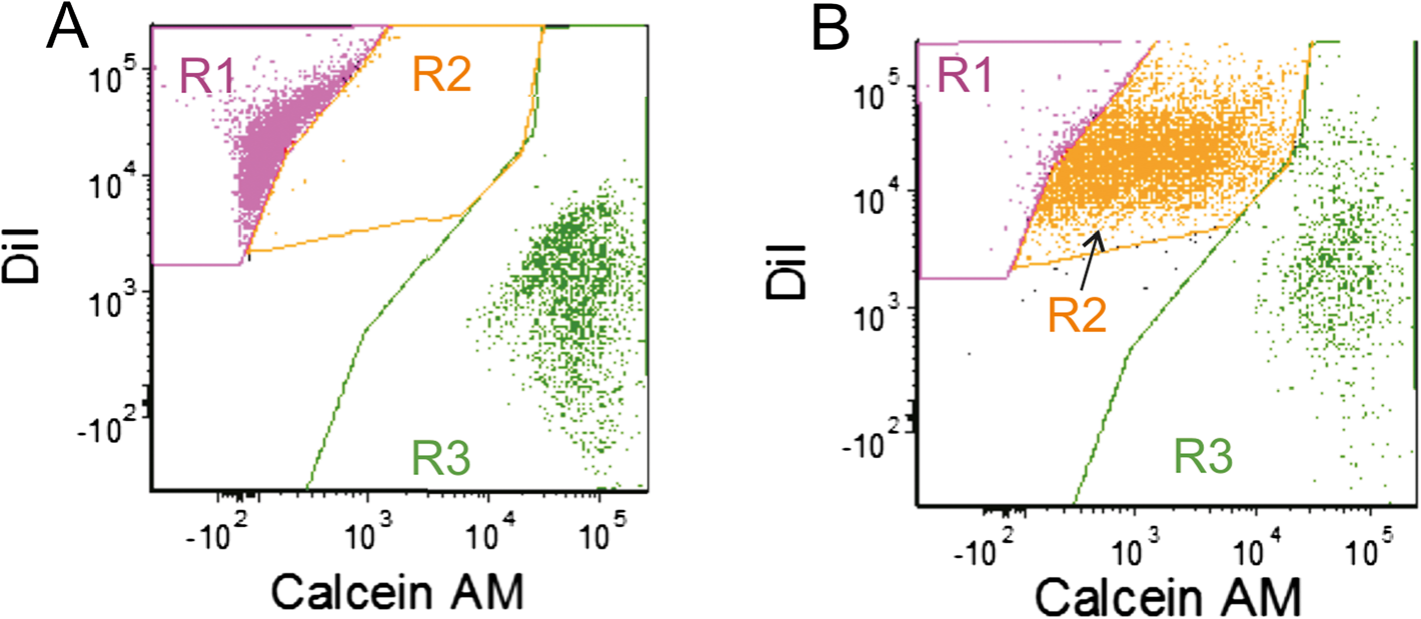
**Evaluation of the functional GJIC between breast cancer-derived T47D cells and AT-MSC based on Calcein AM transfer.** Tumor cells were stained with DiI, AT-MSC were stained with Calcein AM. **A**: T47D cells and AT-MSC were mixed immediately before measurement. **B**: T47D cells and AT-MSC cocultured for 24 h. R1: DiI-positive T47D cells; R2: DiI-positive T47D cell which received Calcein AM from AT-MSC via gap junctions; R3: Calcein AM-positive AT-MSC;

We decided to combine two approaches of MSC-mediated GDEPT in order to improve the efficiency of the treatment. Since the efficacy of the HSVtk/GCV approach depends on a functional intercellular gap-junctional communication (GJIC) [13], only cell lines with functional GJIC (MDA-MB-231, A375 and T47D) were used in this study [[8]; Figure 1].

We performed sequential treatment initially, because we proceeded from the hypothesis that depletion of deoxynucleotide triphosphates (dNTP) pools by cytosine deaminase and 5-FC is important for synergic cooperation of CD/5-FC and HSVtk/GCV approaches [16].

Tumor cells were cultured in the presence of therapeutic cells in various concentrations of prodrug(s), and the mutual cooperation of therapeutic systems was evaluated. The affected fraction (fa; proportion of dead cells) was calculated, dose-effect curves and median-effect plots were created from obtained data. The course of lines in a median-effect plot expressed the mutual interactions between tested therapeutic systems. It was created by plotting *x =* log(D) versus *y =* log(*f*a/*f*u) [D = dose of drug(s); fa = fraction affected (fa = 1- % of viable cells/100 in our case); fu = fraction unaffected (% of viable cells/100). It linearized the sigmoidal dose-effect curves which express the dependence of the effect (fa) on dose of the drug. Parallel lines of median-effect plot indicate that drugs are mutually exclusive, convergent lines mean that systems are mutually non-exclusive, they act in different pathways and affect different targets.

**Figure 2:**
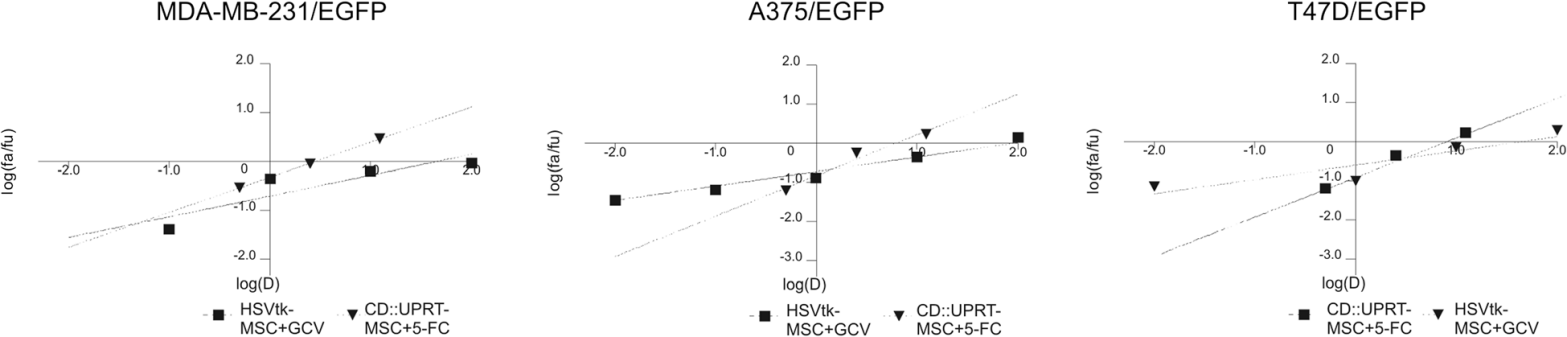
**Median-effect plots demonstrate that cooperation of CD::UPRT/5-FC and HSVtk-MSC/GCV systems is mutually nonexclusive.** EGFP-expressing tumor cells were mixed with CD::UPRT-MSC and HSVtk-MSC in ratio 10:1:1 and treated by 5-FC and/or GCV. Data obtained by fluorimetric assay were subsequently analyzed by Calcusyn software. The particular approaches affect different pathways, because the lines representing the efficacy of CD::UPRT-MSC and HSVtk-MSC approaches are convergent. Log(D) = log of dose – concentration of particular prodrugs; log(fa/fu) = log (proportion of dead cells/proportion of viable cells).

According to the course of lines in median-effect plots, cooperation of CD::UPRT-MSC/5-FC and HSVtk-MSC/GCV systems was evaluated as mutually nonexclusive. Lines expressing the efficacy of particular systems (CD::UPRT/5-FC or HSVtk/GCV) were convergent and thus indicate that active metabolites of 5-FC and GCV affect different pathways (Figure 2).

**Figure 3:**
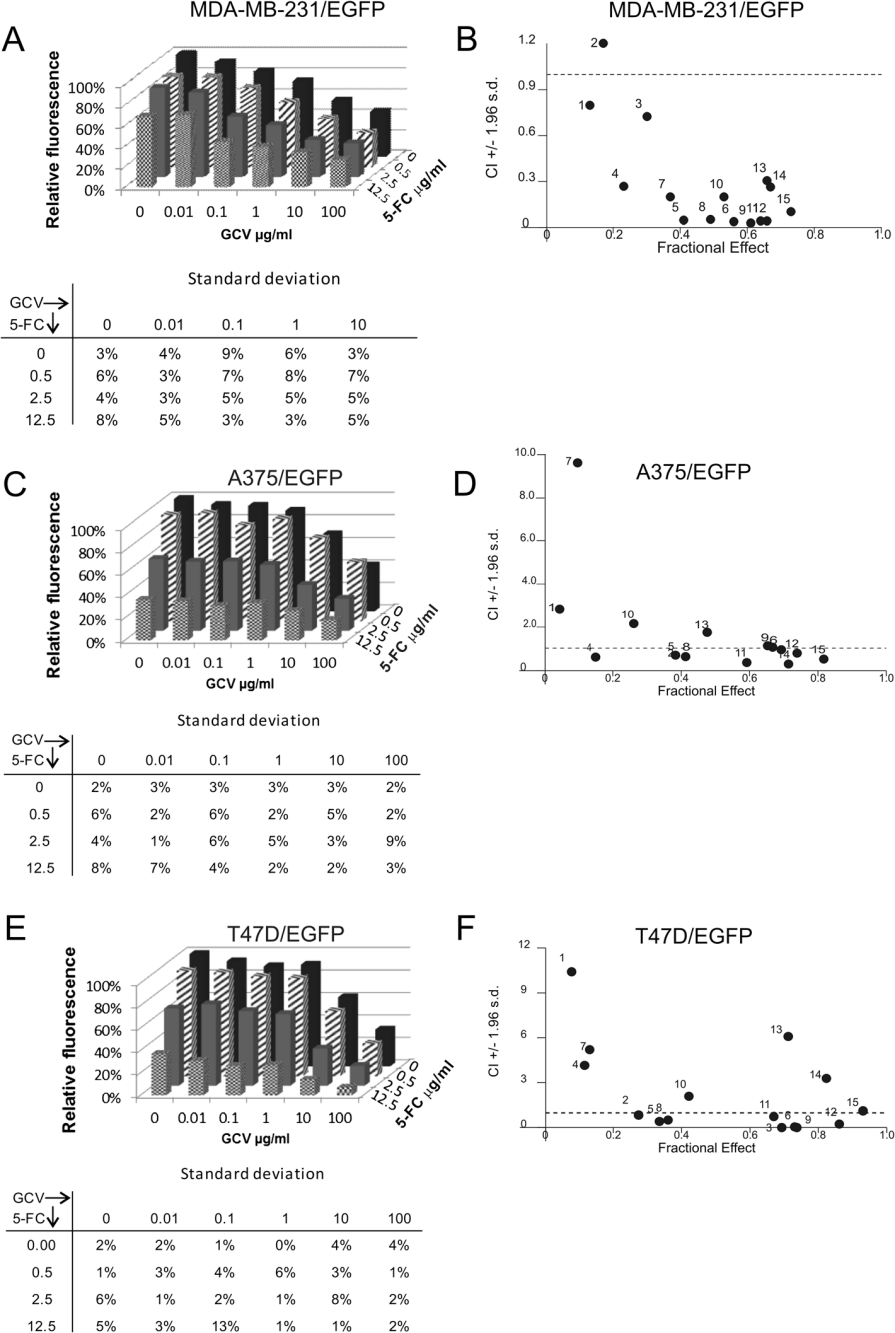
**Synergy of the short-time (72 hrs.) sequential combined enzyme/prodrug treatment** ***in vitro.*** **A**; **C**; **E**: Fluorimetric assay was used for evaluation of the efficacy of the treatment. Relative fluorescence corresponds to the proportion of viable cells. Fluorescence in cocultures without prodrugs was set to 100% by default. Results are presented as mean of quadruplicates. SD are stated in the tables below charts. **B**; **D**; **F**: Data obtained by fluorimetric assay were subsequently analyzed by Calcusyn software, and Fa-CI plots were created – CI (combination index) on the y-axis is a function of effect level (fraction affected, fa) on the x-axis (fa = 1 - % of viable cells/100). Plots display synergism (CI < 1), additivity (CI = 1) or antagonism (CI > 1) for the entire spectrum of effects [14].

We have previously demonstrated the low efficacy of CD::UPRT-MSC/5-FC treatment on MDA-MB-231/EGFP cells [IC_50 5-FC_ = 110 μg/ml as published in [8]] and high sensitivity of this cell line to the HSVtk-MSC/GCV approach [IC_50 GCV_ = 0.06 μg/ml as published in [8]] Despite the low IC_50 GCV_ we were not able to completely eliminate tumor cells from the coculture [8]. In order to improve the cytotoxic effect we applied sequential treatment by 5-FC and GCV on direct coculture of MDA-MB-231/EGFP cells and mixture of CD::UPRT and HSVtk expressing AT-MSC. We observed significant synergy *in vitro* (Figure 3A; B; Additional file 1: Table S1). All of the 15 used combinations of GCV and 5-FC concentrations exerted synergic effect on tumor cells as documented by the value of combination index (CI). The synergy was stronger at higher concentrations (1–100 μg/ml) of GCV (Additional file 1: Table S1).

In order to evaluate if the synergy occurs also in cells responding in opposite manner - sensitive to CD::UPRT-MSC/5-FC and resistant to HSVtk-MSC/GCV treatment, we performed this assay on melanoma-derived A375/EGFP cell line [IC_50 5-FC_ = 3.07 μg/ml; IC_50 GCV_ = 177.02 μg/ml as published in [8]]. The effect was synergic in general, although it was less pronounced in comparison to the MDA-MB-231/EGFP cells (Figure 3C; D; Additional file 1: Table S1).

The breast cancer-derived cell line T47D/EGFP was relatively sensitive to the single enzyme/prodrug treatment (IC_50 5-FC_ = 13.83 μg/ml; IC_50 GCV_ = 3.36 μg/ml) at 72 hours-long treatment (Additional file 2: Figure S1). We observed antagonistic to synergic effect using the identical setup of the experiment. In general, the cooperation of two systems was antagonistic at low concentrations of 5-FC (0.5 μg/ml), and rather synergic at higher concentrations of 5-FC (Figure 3E; F, Additional file 1: Table S1). The statistical analysis of combination indexes (Figure 3B, D and F; Additional file 1: Table S1) by Kruskal-Wallis test showed that there was a significant difference between cell lines used in this study (p = 0.0498). The extent of synergy observed in MDA-MB-231 cells was significantly higher than in A375 cells (p = 0.0086) as evaluated by Mann–Whitney test.

**Figure 4:**
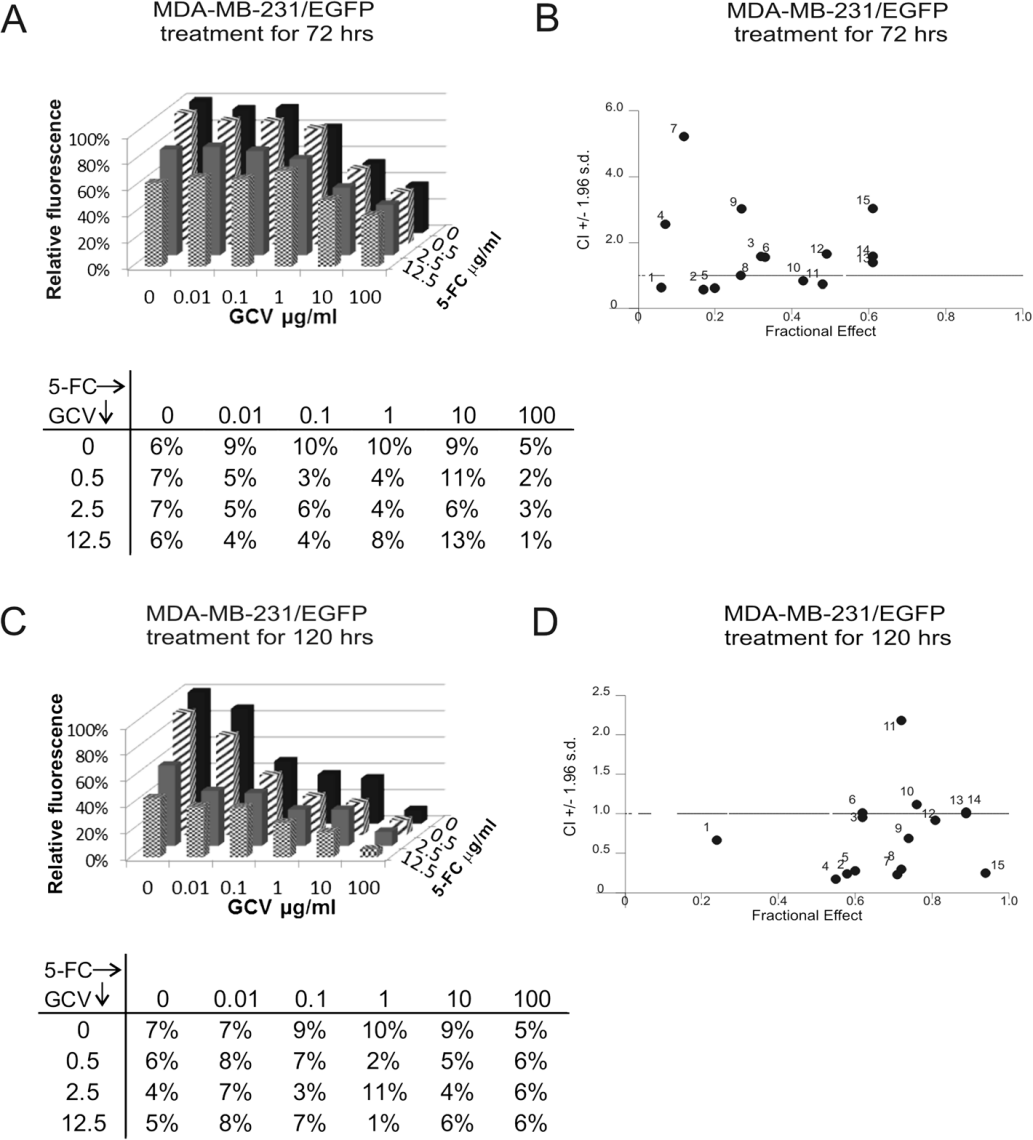
**Effect of the simultaneous combined enzyme/prodrug treatment** ***in vitro.*** **A; C:** Fluorimetric assay was used for evaluation of the efficacy of the treatment. Relative fluorescence corresponds to the proportion of viable cells. Fluorescence in cocultures without prodrugs was set to 100% by default. Results are presented as mean of quadruplicates. SD are stated in the tables below charts. **B**; **D**: Data obtained by fluorimetric assay were subsequently analyzed by Calcusyn software, and Fa-CI plots were created - CI on the y-axis is a function of effect level (fraction affected, fa) on the x-axis (fa = 1 - % of viable cells/100). Plots display synergism (CI < 1), additivity (CI = 1) or antagonism (CI > 1) for the entire spectrum of effects [14].

Since we planned to evaluate the efficacy of the treatment *in vivo*, where it would be difficult to achieve the sequential dosage of particular prodrugs due to their pharmacokinetics, we tested if the synergy occurs also at simultaneous treatment. Contrary to the sequential treatment the synergic effect was suppressed at short-term treatment (Figure 4A-B). The statistical analysis confirmed significant difference (p < 0.0001) in cooperation of two studied systems in short-term sequential and simultaneous setup. The prolonged simultaneous treatment significantly increased the extent of synergy in comparison to short-term (72 hours) treatment (p = 0.0101). The simultaneous treatment seems to be more effective on cell lines A375 and T47D (Additional file 1: Table S1 and Additional file 3: Figure S2), although the differences in cooperation of particular treatments are not significant.

### Systemically administered AT-MSC are able to colonize lungs for short period of time

**Figure 5:**
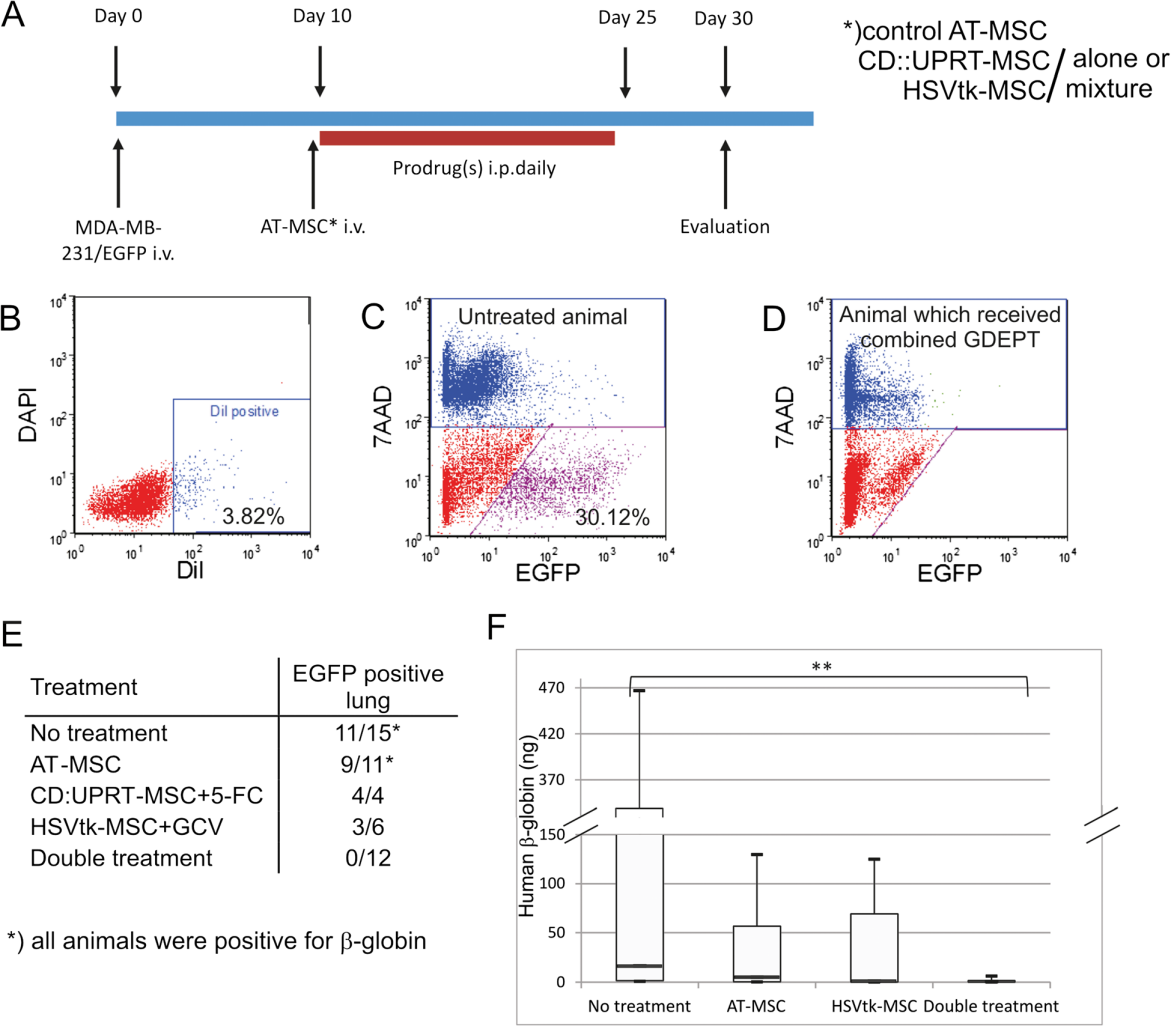
**Efficacy of the treatment** ***in vivo.*** Experimental metastases were induced by intravenous injection of MDA-MB-231/EGFP cells. The presence of human cells in lungs was evaluated by flow cytometry **(B-E)** and by qPCR **(F). A**: Time line of *in vivo* treatment. **B**: Ability of intravenously administered fluorescently labeled AT-MSC to home into mouse lungs. **C**: Presence of the EFGP-positive tumor cells in lung tissue in untreated mouse which received only MDA-MB-231/EGFP cells (an example is presented, we detected 0.28 – 41.6% of viable EGFP-positive cells in mouse lungs, median 4.45). **D**: Absence of EGFP-expressing cells in mouse which received combined GDEPT mediated by engineered AT-MSC. **E**: Summary table of flow cytometric evaluation of the efficacy of individual treatment approaches. **F**: Molecular analysis of lungs. Human β-globin was detected and the amount was calculated according to 150 ng of total DNA. Highly significant difference (p < 0.0001) in amount of human DNA between untreated and double treated animals was detected.

The ability to engraft into the lung tissue is an important factor for interactions between the tumor cells and the therapeutic MSC. We were able to detect fluorescently labelled AT-MSC up to seven days after systemic administration (Figure 5B). Approximately 4% of viable cells were positive for DiI Fluorescent dye.

### Combined treatment by genetically engineered AT-MSC exerts synergy on lung metastases model

We established an experimental breast cancer-derived lung metastases model on SCID/bg mice. Tumor cells were detectable 10 days after the intravenous inoculation by flow cytometry (0.21% of EGFP positive cells) as well as by qPCR (7.97 ng of human DNA/150 ng of total DNA). Flow cytometric analysis showed that 73% (11 out of 15) animals from untreated group and 81% (9 out of 11) mice from group which received untransduced AT-MSC are positive for living, EGFP expressing tumor cells. Molecular analysis confirmed human β-globin specific sequences in all animals in both control groups.

Single as well as combined therapy was applied on experimental tumor-bearing animals (Figure 5A). We did not observe the therapeutic effect of CD::UPRT-MSC/5-FC therapy. The flow cytometric analysis of single cell suspension prepared from lungs of experimental mice revealed that proportion of EGFP-positive cells in lungs of 5-FC treated mice was even higher than in mice which received AT-MSC without subsequent prodrug treatment (median 4.45% vs 13.76% of EGFP positive cells in lungs; Figure 5C).

The treatment with systemically administered HSVtk-MSC and GCV led to decreased occurrence of the tumor cells in lungs (median 0.4% of EGFP-positive cells in lungs). Flow cytometric analysis revealed that three out of six mice were negative for EGFP-expressing MDA-MB-231 cells. Low concentrations of human β-globin sequences were detected by qPCR (median 0.78 ng of human β-globin/150 ng total DNA). Systemically co-administered CD::UPRT-MSC and HSVtk-MSC in combination with simultaneous treatment with 5-FC and GCV prevented proliferation of MDA-MB-231/EGFP cells in mouse lungs. The lungs were analyzed seven to ten days after the finishing of the treatment. None out of 12 experimental animals was positive for EGFP-expressing cells as evaluated by flow cytometry (Figure 5D). Three mice were negative for human β-globin sequences, low amount of human sequences (median 0.38 ng of human β-globin/150 ng total DNA) was detected in rest of the animals in the group.

**Figure 6:**
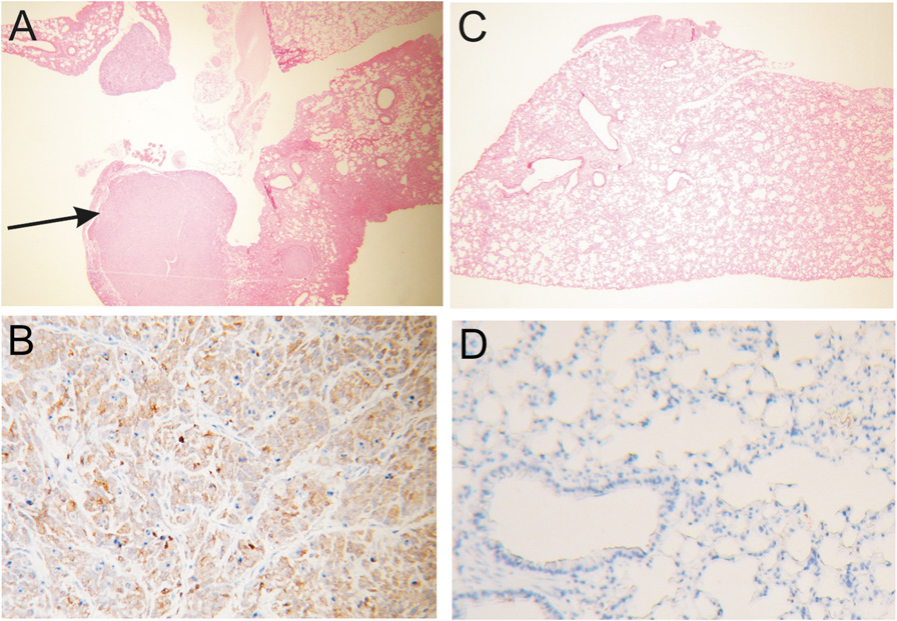
**Histological and immunohistochemical analysis of mouse tissue. A**: Lung section from untreated tumor-bearing animal. Hematoxylin/eosin staining, original magnification × 25. **B**: Detection of EGFP-expressing tumor cells in lung of untreated animal. Immunohistochemical reaction with anti-EGFP polyclonal antibody, original magnification × 200, visualization with 3,3′-diaminobenzidine. **C**: Lung section of animal treated with combined GDEPT. Hematoxylin/eosin staining, original magnification × 25. **D**: No EGFP detection in lung of double treated animal. Immunohistochemical reaction with anti-EGFP polyclonal antibody. Original magnification × 200, visualization with 3,3′-diaminobenzidine.

The summary of flow cytometric analysis is stated in Figure 5E. The double gene therapy mediated by AT-MSC significantly inhibited growth of experimental metastases in mouse lungs (p = 0.0014; Figure 5F). The absence of cancer cells in lungs of double GDEPT-treated animals was confirmed by histological (hematoxylin eosin staining) and immunohistochemical (EGFP detection) staining. (Figure 6A, B, C and D).

## Discussion

The efficacy of GDEPT mediated by MSC derived from various sources has been proven in many preclinical studies. Obstacles connected with (non)viral vectors such as low transgene expression or inefficient penetration into target tissue have been eliminated by the use of MSC or neural progenitors as delivery vehicles. Systemically administered therapeutic MSC were reasonably used for the treatment of many types of tumors [rev. in [17-19]].

On the other hand, we demonstrated that various tumor cell lines differ in the response to CD::UPRT-MSC/5-FC or HSVtk-MSC/GCV treatment depending on the expression of enzymes of nucleotide metabolism and ABC transporters [8,4]. By “classic” approach using (non)viral vectors, it was shown that combined enzyme/prodrug treatment can improve the treatment efficacy [16,20,21].

In various studies, the combination of CD/5-FC and HSVtk/GCV approaches has been described as synergic [16,22] additive [23] or even antagonistic, suggesting negative interactions between CD/5-FC and HSVtk/GCV systems [24].

Slight synergistic cytotoxicity on 9L gliosarcoma cells retrovirally transduced by *E.coli* CD/HSVtk fusion gene was demonstrated by simultaneous treatment of transgene-expressing cells with their specific prodrugs. The toxicity was two-fold higher when expected if the cytotoxic effect of each prodrug was purely additive. Moreover, combined enzyme/prodrug treatment significantly increased the radiosensitivity of tumor cells [25].

Uckert et al. observed additive rather than a synergic effect on CD/HSVtk cotransfected TS/A cells *in vitro*. They supposed that the additive effect could be caused by high sensitivity of TS/A cells to CD/5-FC treatment alone, and addition of HSVtk could only slightly improve the cytotoxic effect [23].

In our study, we used the melanoma cell line A375 which was highly sensitive to CD:UPRT-MSC/5-FC system. The effect of combined treatment was less synergic in comparison to the MDA-MB-231/EGFP cell line, with low sensitivity to the CD::UPRT-MSC/5-FC single treatment. The lower extent of synergy might be caused by high sensitivity to CD::UPRT-MSC/5-FC approach.

Significant synergy was achieved by combined treatment of rat gliosarcoma cell line 9L. Detailed analysis by the isobologram method of Loewe and the multiple drug-effect analysis method of Chou–Talalay revealed cooperation of CD/5-FC and HSVtk/GCV systems in enzymes-expressing cells as well as in the bystander - non-transfected cells. The synergy was proved also on subcutaneous xenotransplants *in vivo* [22].

Boucher et al. observed synergy of combined treatment on prostate carcinoma cells DU145. He put emphasis on sequential treatment, supposing importance of CD/5-FC preincubation leading to alteration in dNTP pools which can modulate the incorporation of phosphorylated GCV into DNA [16].

AT-MSC can home to the tumor and become an integral part of its stromal compartment [rev. in [26]]. This feature provides an opportunity to use them as targeted delivery vehicles for GDEPT [rev.in [19]].

Recently, additive cytotoxic effect of human amniotic fluid-derived stem cells (hAFSC) transfected with bacterial CD::HSVtk fusion gene on MDA-MB-231 cells was described [9]. They observed significant therapeutic effect on athymic mice bearing MDA-MB-231-derived xenografts. In their setup, simultaneous treatment was more efficient in comparison to the sequential 5-FC and GCV treatment, but only one concentration of prodrugs was used for evaluation of the cytotoxicity *in vitro* (5-FC 500 μg/ml, GCV 1 μg/ml), ratio of target/therapeutic cells was 1:2 [9]. We applied gradient concentration of prodrugs and their combination on cocultures consisting of tumor and therapeutic cells in ratio 5:1. Our experiments performed on MDA-MB-231/EGFP cells revealed strong bystander synergy mediated by genetically engineered AT-MSC. Even the synergy was most substantial out of three lines used in our study. The differences in the findings can be caused by different setup of experiments as well as by different suicide genes. Yeast cytosine deaminase is superior to its bacterial counterpart in 5-FC conversion [27]. Moreover, we have used yeast cytosine deaminase fused with uracil phosphoribosyltransferase which even accelerates the conversion of 5-FC to toxic metabolites [28].

In our experimental setup we confirmed observation of Boucher et al. [16] that sequential treatment (single treatment with CD/5-FC followed by HSVtk/GCV) leads to synergic cooperation of CD and HSVtk. We suppose that the final outcome of *in vitro* studies partially depends on the setup of the experiment, duration of the treatment and prodrug concentration. When we used too high concentrations of prodrugs in combination, the synergic effect was suppressed (data not shown).

The sensitivity of tumor cells to the single treatment by CD(::UPRT)/5-FC or HSVtk/GCV probably also contributes to the extent of synergy. We performed double treatment on three tumor cell lines with different sensitivity to the particular approaches, and we observed different response.

Boucher et al. [16] supposed that prolonged administration of prodrugs could contribute to the synergy in simultaneous (concurrent) treatment, as described by Aghi et al. [22], allowing the depletion of dNTPs pools. We demonstrated that prolonged simultaneous treatment led to synergic cooperation of therapeutic approaches. We used long-term simultaneous treatment *in vivo*. We applied both types of therapeutic cells in two injections daily for two weeks. In order to minimize the stress of experimental animals both prodrugs were also administered at the same time. Maximal tolerable doses of prodrugs were administered intraperitoneally.

Two recent papers [10,11] described therapeutic effect of NSC coexpressing CD and HSVtk on tumor cells *in vitro* and *in vivo.* In the later study which focused on the safety of this approach, they demonstrated that therapeutic NSC expressing two prodrug-converting genes showed lower proliferation and viability in comparison to the CD-expressing NSC thus exerting increased safety of this approach. On the other hand, they admitted that therapeutic effect might be limited. As demonstrated in our previous study [8], engineered AT-MSC respond by suicide effect if they are treated with appropriate prodrug. We suppose that the use of the mixture of therapeutic AT-MSC expressing CD::UPRT or HSVtk could prolong the survival of therapeutic MSC and enable to improve the therapeutic effect.

## Conclusion

Although the extent of the synergy of combined GDEPT mediated by MSC depends on the sensitivity of tumor cells to the particular approach and on the experimental setup, cooperation of CD::UPRT-MSC and HSVtk-MSC exerted synergic cytotoxic effect on human breast adenocarcinoma cells MDA-MB-231/EGFP *in vitro*. Systemic combined treatment confirmed the efficacy of this approach by inhibition of proliferation of systemically administered MDA-MB-231/EGFP cells in mouse lungs.

## Additional files

Additional file 1: Table S1. Comparison of the effect of combined treatment on three cells lines used in this study. Additional file 2: Figure S1. Efficacy of the simple enzyme/prodrug treatment by engineered AT-MSC on T47D cells. Cells were cocultured with CD::UPRT-MSC or with HSVtk-MSC for 48 hours. Results are expressed as mean ± SD. **A:** treatment with CD::UPRT-MSC/5-FC. **B:** Treatment with HSVtk-MSC/GCV. Additional file 3: Figure S2. Effect of the simultaneous enzyme/prodrug treatment *in vitro*. Cells were treated for 72 hours (A; B; E; F) or for 120 hours (C; D; G; H). **A; C; E; G:** Fluorimetric assay was used for evaluation of the efficacy of the treatment. Relative fluorescence corresponds to the proportion of viable cells. Fluorescence in cocultures without prodrugs was set to 100% by default. Results are presented as mean of quadruplicates. SD are stated in the tables below charts. **B; D; F; H:** Data obtained by fluorimetric assay were subsequently analyzed by Calcusyn software, and Fa-CI plots were created - CI on the y-axis is a function of effect level (fraction affected, fa) on the x-axis (fa = 1 - % of viable cells/100). Plots display synergism (CI < 1), additivity (CI = 1) or antagonism (CI > 1) for the entire spectrum of effects [14].

## Abbreviations

5-FC: 5-fluorocytosine
5-FU: 5-fluorouracil
AT-MSC: Adipose tissue-derived mesenchymal stromal cells
CD: Cytosine deaminase
CD: UPRT: Cytosine deaminase fused with uracil phosphoribosyltransferase
CI: Combination index
fa: Fraction affected
fu: Fraction unaffected
GJIC: Gap-junctional intercellular communication
HSVtk: *Herpes simplex virus* thymidine kinase
NSC: Neural stem cells
