# Supplementary material for: Combined enzyme/prodrug treatment by genetically engineered AT-MSC exerts synergy and inhibits growth of MDA-MB-231 induced lung metastases
Source: J Exp Clin Cancer Res. 2015 Apr 9;34(1):33. doi: 10.1186/s13046-015-0149-2 (PMC4431639; doi:10.1186/s13046-015-0149-2)
Supplement: Additional file 1: Table S1. — Comparison of the effect of combined treatment on three cells lines used in this study. [file 13046_2015_149_MOESM1_ESM.docx]

**Additional table 1:** Comparison of the effect of combined treatment on three cells lines used in this study

Data obtained by fluorimetric assay were analyzed

Fa – fraction affected (fa = 1- % of viable cells /100)

CI – combination index [synergism (CI<1), additivity (CI=1), antagonism (CI>1)]

|  |  |  | **Sequential treatment (5-FC for 24 hrs.+ GCV for 48 hrs.)** | | | | | | **Simultaneous treatment for 72 hrs.** | | | | | | **Simultaneous treatment for 120 hrs.** | | | | | |
| --- | --- | --- | --- | --- | --- | --- | --- | --- | --- | --- | --- | --- | --- | --- | --- | --- | --- | --- | --- | --- |
| **No.** | **GCV (µg/ml)** | **5-FC (µg/ml)** | **MDA-MB-231** | | **A375** | | **T47D** | | **MDA-MB-231** | | **A375** | | **T47D** | | **MDA-MB-231** | | **A375** | | **T47D** | |
|  |  |  | **Fa** | **CI** | **Fa** | **CI** | **Fa** | **CI** | **Fa** | **CI** | **Fa** | **CI** | **Fa** | **CI** | **Fa** | **CI** | **Fa** | **CI** | **Fa** | **CI** |
| 1 | 0.01 | 0.50 | 0.13 | 0.82 | 0.04 | 2.88 | 0.08 | 10.65 | 0.06 | 0.629 | 0.1 | 1.523 | 0.31 | 1.421 | 0.24 | 0.773 | 0.17 | 0.931 | 0.51 | 0.715 |
| 2 | 0.01 | 2.50 | 0.17 | 1.21 | 0.38 | 0.65 | 0.27 | 0.87 | 0.17 | 0.587 | 0.41 | 0.928 | 0.62 | 0.634 | 0.58 | 0.243 | 0.47 | 1.192 | 0.78 | 0.641 |
| 3 | 0.01 | 12.50 | 0.30 | 0.73 | 0.65 | 1.13 | 0.69 | 0.03 | 0.32 | 1.589 | 0.71 | 1.285 | 0.74 | 1.216 | 0.62 | 0.966 | 0.89 | 1.249 | 0.9 | 0.987 |
| 4 | 0.10 | 0.50 | 0.23 | 0.29 | 0.15 | 0.64 | 0.11 | 3.80 | 0.07 | 2.732 | 0.23 | 0.86 | 0.57 | 0.495 | 0.55 | 0.188 | 0.27 | 1.001 | 0.67 | 0.718 |
| 5 | 0.10 | 2.50 | 0.41 | 0.05 | 0.38 | 0.66 | 0.33 | 0.44 | 0.2 | 0.657 | 0.46 | 0.831 | 0.69 | 0.509 | 0.60 | 0.302 | 0.58 | 0.897 | 0.79 | 0.807 |
| 6 | 0.10 | 12.50 | 0.56 | 0.04 | 0.69 | 0.94 | 0.74 | 0.02 | 0.33 | 1.591 | 0.77 | 0.945 | 0.75 | 1.233 | 0.62 | 1.073 | 0.91 | 1.068 | 0.91 | 0.911 |
| 7 | 1 | 0.50 | 0.37 | 0.20 | 0.10 | 9.44 | 0.13 | 5.47 | 0.12 | 5.779 | 0.42 | 0.946 | 0.75 | 0.707 | 0.71 | 0.243 | 0.33 | 3.035 | 0.86 | 0.826 |
| 8 | 1 | 2.50 | 0.49 | 0.05 | 0.41 | 0.63 | 0.36 | 0.63 | 0.267 | 1.009 | 0.54 | 0.978 | 0.77 | 0.8 | 0.72 | 0.323 | 0.66 | 0.805 | 0.9 | 0.679 |
| 9 | 1 | 12.50 | 0.61 | 0.03 | 0.67 | 1.06 | 0.73 | 0.08 | 0.27 | 3.134 | 0.75 | 1.161 | 0.86 | 0.568 | 0.74 | 0.721 | 0.93 | 0.883 | 0.91 | 1.475 |
| 10 | 10 | 0.50 | 0.53 | 0.20 | 0.26 | 2.65 | 0.42 | 2.14 | 0.43 | 0.876 | 0.7 | 0.974 | 0.93 | 0.438 | 0.76 | 1.145 | 0.6 | 1.751 | 0.95 | 1.345 |
| 11 | 10 | 2.50 | 0.64 | 0.05 | 0.59 | 0.34 | 0.67 | 0.76 | 0.48 | 0.785 | 0.72 | 1.163 | 0.93 | 0.459 | 0.72 | 2.217 | 0.82 | 0.505 | 0.95 | 1.49 |
| 12 | 10 | 12.50 | 0.66 | 0.04 | 0.74 | 0.77 | 0.86 | 0.25 | 0.49 | 1.847 | 0.87 | 0.673 | 0.9 | 1.207 | 0.81 | 0.993 | 0.95 | 0.691 | 0.96 | 1.484 |
| 13 | 100 | 0.50 | 0.66 | 0.31 | 0.48 | 1.74 | 0.71 | 6.13 | 0.61 | 1.435 | 0.87 | 1.466 | 0.96 | 1.49 | 0.89 | 1.006 | 0.85 | 0.663 | 0.99 | 1.09 |
| 14 | 100 | 2.50 | 0.67 | 0.26 | 0.72 | 0.28 | 0.82 | 3.28 | 0.61 | 1.699 | 0.91 | 0.802 | 0.98 | 0.413 | 0.89 | 1.054 | 0.9 | 0.46 | 0.99 | 1.108 |
| 15 | 100 | 12.50 | 0.73 | 0.10 | 0.82 | 0.51 | 0.93 | 1.13 | 0.61 | 3.021 | 0.9 | 1.492 | 0.94 | 3.51 | 0.94 | 0.265 | 0.96 | 0.61 | 0.98 | 3.643 |
|  |  | Synergy  Antagonism   \|  \| \| --- \| |  |  |  |  |  |  |  | Additive effect |  |  |  |  |  |  |  |  |  |  |
|  |  |  |  |  |  |  |  |  |  |  |  |  |  |  |  |  |  |  |  |  |
