# Supplementary figures and images for: Combined enzyme/prodrug treatment by genetically engineered AT-MSC exerts synergy and inhibits growth of MDA-MB-231 induced lung metastases
Source: J Exp Clin Cancer Res. 2015 Apr 9;34(1):33. doi: 10.1186/s13046-015-0149-2 (PMC4431639; doi:10.1186/s13046-015-0149-2)

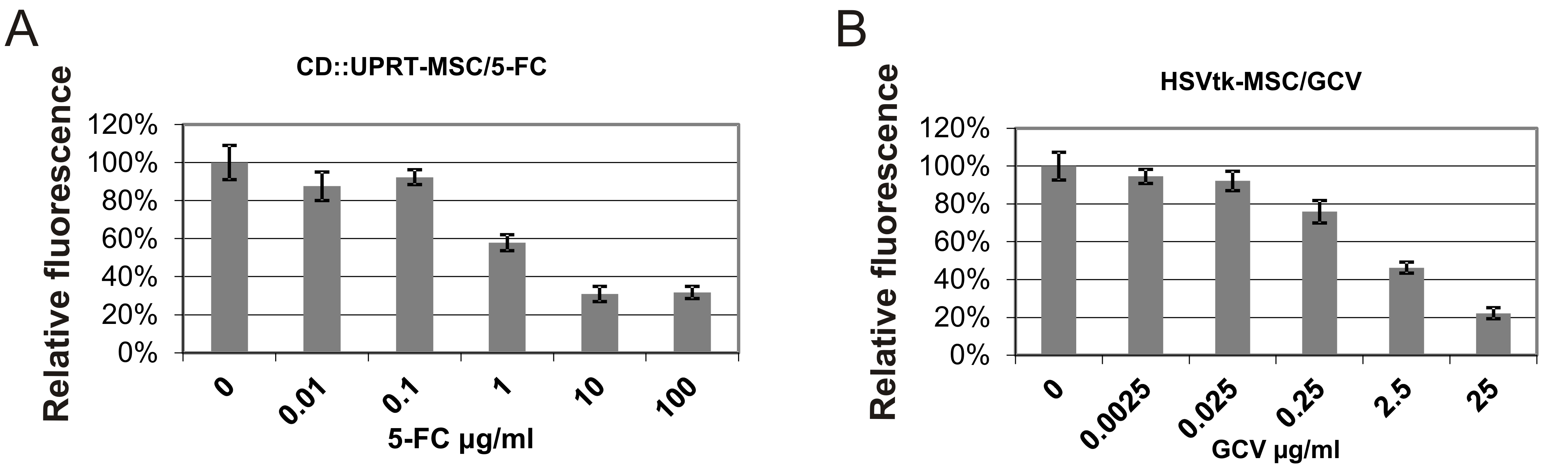

Supplement: Additional file 2: Figure S1. — Efficacy of the simple enzyme/prodrug treatment by engineered AT-MSC on T47D cells. Cells were cocultured with CD::UPRT-MSC or with HSVtk-MSC for 48 hours. Results are expressed as mean ± SD. A: treatment with CD::UPRT-MSC/5-FC. B: Treatment with HSVtk-MSC/GCV. [file 13046_2015_149_MOESM2_ESM.tif]
